# Detection of *Japanese Encephalitis Virus* RNA in Human Throat Samples in Laos – A Pilot study

**DOI:** 10.1038/s41598-018-26333-4

**Published:** 2018-05-22

**Authors:** Tehmina Bharucha, Onanong Sengvilaipaseuth, Malee Seephonelee, Malavanh Vongsouvath, Manivanh Vongsouvath, Sayaphet Rattanavong, Géraldine Piorkowski, Marc Lecuit, Christopher Gorman, Jean-David Pommier, Paul N. Newton, Xavier de Lamballerie, Audrey Dubot-Pérès

**Affiliations:** 1 Lao-Oxford-Mahosot Hospital-Wellcome Trust Research Unit (LOMWRU), Microbiology Laboratory, Mahosot Hospital, Vientiane, Lao PDR; 2 Division of Infection and Immunity, University College London, London, UK; 3 Centre for Tropical Medicine and Global Health, Nuffield Department of Clinical Medicine, University of Oxford, Churchill Hospital, Oxford, UK; 4 UMR “Unité des Virus Emergents” (UVE: Aix-Marseille Univ – IRD 190 – Inserm 1207 – IHU Méditerranée Infection), Marseille, France; 5 Institut Pasteur, Biology of Infection Unit, Inserm, U1117 Paris, France; 6 Paris Descartes University, Necker-Enfants Malades University Hospital, Division of Infectious Diseases and Tropical Medicine, Paris, France; 7 Institut Pasteur du Cambodge, Institut Pasteur International Network, Phnom Penh, Cambodia

## Abstract

*Japanese encephalitis virus* (JEV) is the most commonly identified cause of acute encephalitis syndrome (AES) in Asia. The WHO recommended test is anti-JEV IgM-antibody-capture-enzyme-linked-immunosorbent-assay (JEV MAC-ELISA). However, data suggest this has low positive predictive value, with false positives related to other *Flavivirus* infections and vaccination. JEV RT-PCR in cerebrospinal fluid (CSF) and/or serum is highly specific, but is rarely positive; 0–25% of patients that fulfil the WHO definition of JE (clinical Acute Encephalitis Syndrome (AES) and JEV MAC-ELISA positive). Testing other body fluids by JEV RT-qPCR may improve the diagnosis. As a pilot study thirty patients admitted to Mahosot Hospital 2014–2017, recruited to the South-East-Asia-Encephalitis study, were tested by JEV MAC-ELISA and two JEV real-time RT-PCR (RT-qPCR) assays (NS2A and NS3). Eleven (36.7%) were JEV MAC-ELISA positive. Available CSF and serum samples of these patients were JEV RT-qPCR negative but 2 (7%) had JEV RNA detected in their throat swabs. JEV RNA was confirmed by re-testing, and sequencing of RT-qPCR products. As the first apparent report of JEV RNA detection in human throat samples, the provides new perspectives on human JEV infection, potentially informing improving JEV detection. We suggest that testing patients’ throat swabs for JEV RNA is performed, in combination with molecular and serological CSF and serum investigations, on a larger scale to investigate the epidemiology of the presence of JEV in human throats. Throat swabs are an easy and non-invasive tool that could be rolled out to a wider population to improve knowledge of JEV molecular epidemiology.

## Introduction

Evidence continues to implicate *Japanese encephalitis virus* (JEV) as a major cause of encephalitis in Asia, with recent evidence of possible autochthonous transmission in Africa^1–3^. Among the key factors in its persistent role as a public health problem are the limitations of existing diagnostic tests, our understanding of its epidemiology and inadequate implementation of vaccination programmes.

The conventional mainstay of JEV encephalitis diagnosis is serology, with serum and cerebrospinal fluid (CSF) anti-JEV IgM antibody capture enzyme-linked immunosorbent assays (JEV MAC-ELISA) recommended by the World Health Organization (WHO)^4^. However, there are concerns about the accuracy of JEV MAC-ELISA for diagnosing JEV. Low positive predictive value of JEV MAC-ELISA has been reported^5^, and there are recognised difficulties with false positives related to vaccination and in areas where other *Flavivirus* infections are endemic^6,7^.

Diagnosis of JEV by Reverse-Transcription (RT)-PCR is highly specific, and enables improved understanding of the molecular epidemiology of JEV^8^. However, JEV RT-PCR testing of CSF and serum samples has low sensitivity, rarely positive in patients at presentation (0–25% that fulfil the WHO definition of Acute Encephalitis Syndrome (AES) and JEV MAC-ELISA positivity)^5,9^. Other body fluids may provide additional source of JEV RNA but there has been little investigation of this possibility. Other Flaviviruses, such as *West Nile virus*, *Zika virus* and *Dengue virus*, have been detected in urine, and JEV RNA has recently been detected in the urine of two patients^10–13^.

Human throat swab samples have apparently not been used to test for JEV RNA, although there is recent evidence of high JEV viral loads in the oronasal secretions in the pigs, the amplifying host, and that the tonsils may be a site of viral replication and contribute to transmission^14^.

We therefore hypothesised that JEV may be present in the throats of human patients. If this is the case, the use of non-invasive throat swab clinical samples could potentially improve the diagnosis of JEV and our understanding of JEV molecular epidemiology. We performed a pilot study to evaluate if JEV RNA could be detected by RT-qPCR in throat swabs of patients admitted to Mahosot Hospital, Vientiane, Laos, with AES clinical presentation.

## Results

From November 2014 to June 2017, 129 patients admitted to Mahosot Hospital who had lumbar puncture presented with clinical AES presentation. Of these, 61 patients presented within 7 days of disease onset and 40 of these were anti-JEV IgM positive on CSF and/or serum or undiagnosed following routine testing. Throat swab samples from 30 patients were available and were included in this study. Eleven (36.7%) patients were anti-JEV IgM MAC-ELISA positive in CSF and admission serum (Table 1). CSF samples were available for 9 patients and sera from 6 patients. All CSF and serum samples were negative by JEV RT-qPCR, but 2 (7%) patients had JEV RNA detected in their throat swabs, patient 5 and patient 10 (Table 1). Anti-JEV IgM was detected in CSF and admission sera from both patients.

**Table 1:** Patients with positive results for JE MAC-ELISA or JEV RT-qPCR.

Patient number	Age (yr)	Sex	JE MAC-ELISA (ISR)°			JEV RT-qPCR (Ct value)					
			Admission Serum	Convalescent Serum	CSF	Throat swab		CSF		Serum	
						**NS2A**	**NS3**	**NS2A**	**NS3**	**NS2A**	**NS3**
1	19	M	Pos (18.4)	Pos (45.1)	Pos (29.8)	Neg	Neg	Neg	Neg	Neg	Neg
2	50	M	Pos (26.0)	Pos (40.8)	Pos (34.2)	Neg	Neg	Neg	Neg	Neg	Neg
3	33	M	Pos (40.6)	Neg (3.2)	Pos (35.1)	Neg	Neg	Neg	Neg	—	—
4	7	M	Pos (17.7)	—	Pos (44.4)	Neg	Neg	Neg	Neg	—	—
5	16	M	Pos (7.9)	Pos (18.0)	Pos (21.6)	Pos (32)	Pos (37)	Neg	Neg	—	—
6	13	M	Pos (11.3)	Pos (27.9)	Pos (60.3)	Neg	Neg	Neg	Neg	—	—
7	14	M	Pos (21.7)	—	Pos (38.2)	Neg	Neg	Neg	Neg	Neg	Neg
8	3	F	Pos (13.6)	—	Pos (33.9)	Neg	Neg	—	—	Neg	Neg
9	3	M	Pos (18.0)	—	Pos (41.8)	Neg	—	Neg	—	Neg	—
10	13	M	Pos (22.3)	—	Pos (42.8)	Pos (36)	—	Neg	—	Neg	—
11	23	M	Pos (6.3)	Neg (4.8)	Pos (6.4)	Neg	Neg	—	—	—	—

M: male, F: female. CSF: cerebrospinal fluid. °Anti-JEV IgM detection using JE Detect™ IgM Antibody Capture ELISA Kit (InBios, Washington, USA). Positive (Pos) = Anti-JEV IgM positive, negative (Neg) = Anti-JEV IgM negative or equivocal, with ISR (Immune status Ratio) cut-offs calculated according to the manufacturer’s instructions. RT-qPCR negative (Neg) = no amplification curve or curve with a Cq > 40. RT-qPCR positive (Pos) = amplification curve with Cq < 40. “−“ = no sample available for testing due to small sample volume collected and/or the use in its entirety for previous assays.

For patient 5, both NS2A and NS3 RT-qPCRs from the throat swab were positive (Ct 32 and 37, respectively). This throat swab was re-extracted and retested, and results demonstrated a NS2A RT-qPCR Ct of 34 and a negative NS3 RT-qPCR. For patient 10, only NS2A RT-qPCR was performed from the throat swab and was found positive with a Ct of 36. Unfortunately, sample volume was insufficiency to permit repetition or NS3 RT-qPCR.

**Figure 1:**
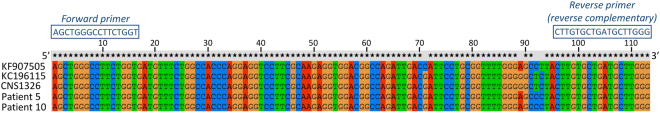
Sequences of the amplicons obtained by NS2A JEV RT-qPCR from throat swabs of patients 5 and 10. Sequences obtained for the 2 patients were aligned (using ClustalX 2.1) with the corresponding sequences of all JEV strains ever handled in the laboratory. KF907505: JEV RNA used as positive control for all JEV RT-qPCR runs. KC196115 and CNS1326: the two JEV strains isolated on cell culture from CSF patients in 2009 and 2013 respectively. Sequence identities are indicated by stars. Sequences of the forward and reverse primers used for the JEV RT-qPCR are indicated above the sequence alignment.

Sequences of NS2A RT-qPCR products for patients 5 and 10 are displayed in Fig. 1. BLAST search on the NCBI website (blastn) showed 100% identities with described JEV isolates. Sequence alignment (Fig. 1) shows two nucleotide differences (C/G at position 71 and T/C at position 93) with the sequence of the JEV RNA used as positive control. We also compared the NS2A sequences from patients 5 and 10 with the two JEV strains that were isolated for patients CSF in 2009 and 2013 and observed two nucleotide differences (G/A at position 89 and T/C at position 92).

## Discussion

We provide strong evidence of JEV RNA detection in throat swab from two patients with clinical presentation of AES. Sequencing of JEV RT-qPCR products confirmed the detection of JEV sequences with nucleotide differences as compared to all JEV strains handled in the laboratory, reducing the likelihood of contamination having occurred. JEV RNA was detected from patient 5 using two different RT-qPCR systems targeting two different genes (NS2A and NS3) and in two sample aliquots from same patient extracted at different time.

Consistent with the literature that JEV RNA is rarely detected in CSF or serum samples, neither of the throat swab JEV positive patients had JEV RNA detected in CSF or serum. Therefore, the use of throat swabs in addition to serum and CSF increased JEV molecular detection. In contrast to JEV MAC-ELISA, the detection of JEV RNA is highly specific for confirming JEV infection. Throat swabs are non-invasive samples, which may be useful to improve diagnosis, especially in patients in whom a lumbar puncture (LP) is not possible or where LP facilitates are not available. Throat swab collection does not require specific facilities, and therefore could facilitate sampling from a wider population to improve our understanding of JEV molecular epidemiology.

There are minimal data on the detection of JEV RNA in other body fluids of patients. One retrospective study did not detect JEV RNA in urine of 52 patients in China^15^. More recently, JEV RNA has been detected in the urine of two patients^10,13^. It is likely that JEV RNA may be excreted in urine intermittently and at low levels. We are not aware of any published studies testing for JEV RNA in human throat swabs. However, our findings are consistent with the study by Ricklin *et al*., in pigs (14). However, the current study was performed on only a small number patients’ samples, and larger studies are needed to better understand the role of throat samples in the detection of JEV RNA by RT-PCR, especially on the frequency and duration of throat swab positivity in relation to clinical presentation, anti-JEV IgM and CSF positivity and patient age.

Our findings also raise the question of possible throat excretion of live JEV virus in infected patients. Throat swab samples from patients 5 and 10 were inoculated on Vero and C6/36 cells but unfortunately the virus could not be isolated, probably due to the low virus titre, but we cannot exclude that JEV RNA fragments alone were present in the throat. More extensive studies are required to investigate the epidemiology of live JEV in human throats.

## Methods

### Patients

We performed a retrospective study of patients recruited in the South-East Asia Encephalitis study^16^ conducted at Mahosot Hospital, Vientiane, Laos, between November 2014 to June 2017. CSF, serum and throat swab (using Sigma Virocult®, Medicale Wire, Wiltshire, England) samples at patient inclusion, and follow-up serum at hospital discharge, were collected and stored at −80 °C for subsequent testing. Patients included in our study met all the following criteria: (i) were admitted to hospital with sings/symptoms fulfilling the WHO clinical case definition for AES^4^, (ii) had no contraindication for lumbar puncture, (iii) presented within 7 days of onset of symptoms, (iv) gave written informed consent, (v) were found anti-JEV IgM positive or undiagnosed following routine diagnosis (vi) had throat swabs available for testing.

Ethical clearance was granted by the Ethical Review Committee of the Faculty of Medical Sciences, National University of Lao, and the Oxford University Tropical Ethics Research Committee, Oxford, UK. The study was performed in accordance with relevant guidelines and regulations.

### Anti-JEV IgM ELISA (JE MAC-ELISA)

A WHO recommended commercial JE MAC-ELISA assay is the InBios JE Detect kit (Washington, USA)^17^. As hospital routine diagnosis procedure, CSF and serum samples from patients with suspicion of CNS infections were tested using this kit following manufacturer’s instruction. A 1/10 dilution was used for CSF samples as recommended by WHO. For each tested sample, ISR (Immune Status Ratio) was calculated and interpreted for the qualitative detection of anti-JEV IgM following manufacturer’s instructions: positive if >6.0, equivocal if 4.0–6.0, or negative if <4.0.

### RNA extraction

After defrosting, 200 µL of each sample was extracted and eluted in 60 µL using EZ1 Virus Mini Kit v2.0 (Qiagen, Hilden, Germany) following manufacturer’s instruction.

### Real-time RT-PCR (RT-qPCR)

Two in-house JEV RT-qPCR assays were performed, targeting different parts of the JEV genome, NS2A and NS3. Primers and probes were designed to identify the best in-silico matching from a multiple genome alignment of all JEV sequences (303) above 9,000 base pairs available on GenBank. Optimisation and validation assays (Bharucha *et al*., in prep., Supplementary Data Fig. 1) show good analytical sensitivity (approximately 4 copies/reaction) and 100% specificity when tested on a set of patients and on other viruses from JEV serocomplex. NS2A RT- qPCR was performed with TaqMan® Fast Virus 1-Step kit (Thermo Fisher, Waltham, USA): 50 μL reaction volume; 600 nM forward (5′-AGCTGGGCCTTCTGGT-3′) and reverse (5′-CCCAAGCATCAGCACAAG-3′) primers; 300 nM probe (5′ FAM-CTTCGCAAGAGGTGGACGGCCA-TAMRA 3′) and 30 μL RNA. Thermocycling conditions: 50 °C for 5 minutes, 95 °C for 20 seconds, 45 × [95 °C for 15 seconds +62 °C for 60 seconds]. NS3 RT-qPCR was performed with SuperScript™ III One-Step RT-PCR System with Platinum™ Taq DNA Polymerase kit (Thermo Fisher, Waltham, USA): 50 μL reaction volume; 600 nM forward (5′-GCAATGTGYCTCCAAAGAGC-3′) and reverse (5′-GTCGATGACCCTGCTCGC-3′) primers; 300 nM probe (5′FAM-TCCTATGAYACAGAATAYCCAAA-MGB NFQ 3′); 15 μL RNA. Thermocycling conditions: 50 °C for 15 minutes; 95 °C for 2 minutes; 45 × [95 °C for 15 seconds +56 °C for 45 seconds]. RNA positive control (UVE/JEV/UNK/TW/RP9-190 strain, GenBank KF907505, EVA 001V-02344) and no template control were also included to each RT-qPCR run. JEV RT-qPCR result with Ct < 40 was classified as positive.

Due to limitation in sample volume available, NS2A RT- qPCR was performed first, then NS3 when sample still available. In case of any positive the corresponding RT-qPCR was repeated on new extraction, when sample available.

An internal control (MS2 phage) was added to each patient sample prior to extraction. MS2 RT-qPCR was performed to control the extraction process and to exclude inhibition as previously described^18^.

### Sequencing

When available, positive RT-qPCR results were further investigated by sending the RT-qPCR product for next-generation sequencing using Ion S5 system (Thermo Fisher, Waltham, USA), to Unité des Virus Emergents, Faculty of Medicine, Marseille, France.

Sequences were aligned, using ClustalX 2.1^19^, with the sequence of the positive control used for the JEV RT-qPCR and the sequences of any other JEV RNA handled in the laboratory.

### Data availability

Every effort has been made to present the data generated and analysed during the study in entirety. The corresponding author will be able to provide further details on reasonable request.

## Electronic supplementary material

Supplementary Information
