# Supplementary material for: Detection of Japanese Encephalitis Virus RNA in Human Throat Samples in Laos – A Pilot study
Source: Sci Rep. 2018 May 22;8:8018. doi: 10.1038/s41598-018-26333-4 (PMC5964078; doi:10.1038/s41598-018-26333-4)

**Supplementary Data**

**Detection of *Japanese Encephalitis Virus* RNA in Human Throat Samples in Laos – A Pilot study**

Tehmina Bharucha^1,2*^, Onanong Sengvilaipaseuth^1^, Malee Seephonelee^1^, Malavanh Vongsouvath^1^, Manivanh Vongsouvath^1^, Sayaphet Rattanavong^1^, Géraldine Piorkowski^4^, Marc Lecuit^5,6^, Christopher Gorman^7^, Jean-David Pommier^7^, Paul N Newton^1,3^, Xavier de Lamballerie^4^, Audrey Dubot-Pérès^1,3,4^

^1.^ Lao-Oxford-Mahosot Hospital-Wellcome Trust Research Unit (LOMWRU), Microbiology

Laboratory, Mahosot Hospital, Vientiane, Lao PDR

^2.^ Division of Infection and Immunity, University College London, London, UK

^3.^ Centre for Tropical Medicine and Global Health, Nuffield Department of Clinical Medicine, University of Oxford, Churchill Hospital, Oxford, UK

^4.^ UMR "Unité des Virus Emergents" (UVE: Aix-Marseille Univ – IRD 190 – Inserm 1207 – IHU Méditerranée Infection), Marseille, France

^5.^ Institut Pasteur, Biology of Infection Unit, Inserm U1117, Paris, France

^6.^ Paris Descartes University, Necker-Enfants Malades University Hospital, Division of Infectious Diseases and Tropical Medicine, Paris, France

^7.^ Institut Pasteur du Cambodge, Institut Pasteur International Network, Phnom Penh, Cambodia

*Corresponding author: Dr Tehmina Bharucha, [t.bharucha@doctors.org.uk](mailto:t.bharucha@doctors.org.uk). Lao-Oxford-Mahosot Hospital-Wellcome Trust Research Unit (LOMWRU), Microbiology Laboratory, Mahosot Hospital, Vientiane, Lao PDR

**S1 Fig: Standard curves of the 1) NS2A real-time RT-PCR assay and 2) NS3 real-time RT-PCR assay using serial dilutions of RNA positive control on (A) Day 1 and repeated on (B) Day 2.** Result of the RT-qPCR run, ‘Cq’, is plotted against the ‘log starting quantity’, at the RNA dilutions tested: NS2A assay 1:10 serial dilutions of G1-769 (GenBank KC196115, EVA 001V-02217) in triplicate at 10^-3^ to 10^-7^; and NS3 with G3-RP-190 (GenBank KF907505, EVA 001V-02344) 10^-4^ to 10^-7^. Efficiency=10^-1/slope^-1. R^2^=Correlation Coefficient. RT-qPCR performed with Fastvirus kit (TaqMan® Fast Virus 1-Step) with a reaction volume of 50µL, sample volume of 30µl, and primer and probe concentrations of 600nM and 300nM respectively. Thermocycling conditions were 50°C for 5 minutes, 95°C for 20 seconds and 45 x (95°C for 15 seconds + x°C for 60 seconds). The annealing temperature ‘x°C’ was 60°C and 56°C for the NS2A and NS3 assays respectively.

**2A. NS2A Day 1 2B. NS2A Day 2**


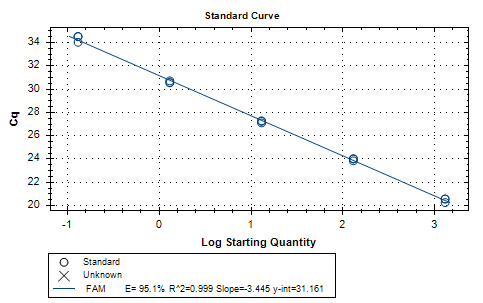

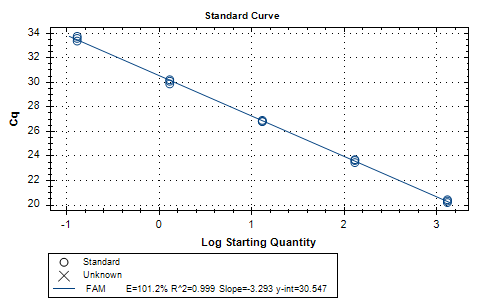


**3A. NS3 Day 1 3B. NS3 Day 2**


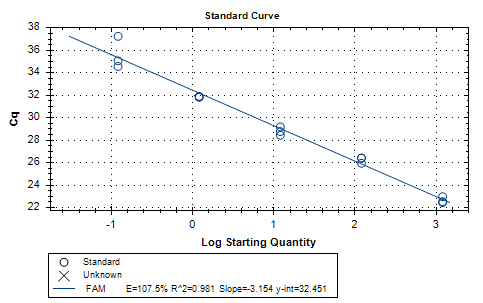

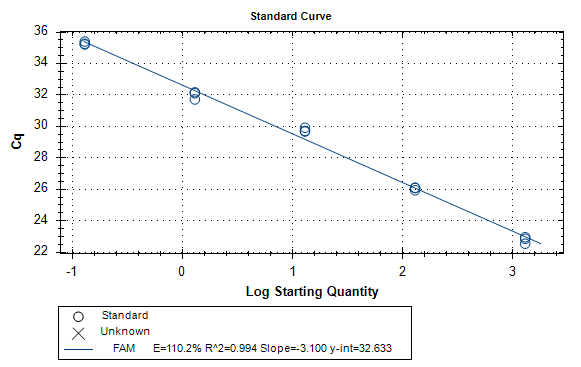

Supplement: Supplementary file 1 — Supplementary Information [file 41598_2018_26333_MOESM1_ESM.docx]
